# Palmaris Longus Muscle’s Prevalence in Different Nations and Interesting Anatomical Variations: Review of the Literature

**DOI:** 10.14740/jocmr2243w

**Published:** 2015-09-25

**Authors:** Dimitriou Ioannis, Katsourakis Anastasios, Natsis Konstantinos, Kostretzis Lazaros, Noussios Georgios

**Affiliations:** a Laboratory of Anatomy in the Department of Physical Education and Sports Medicine (Serres), Aristotele University of Thessaloniki, Thessaloniki, Greece; b Department of Surgery, “Agios Dimitrios” General Hospital of Thessaloniki, Thessaloniki, Greece; c Department of Anatomy, Medical School, Aristotele University of Thessaloniki, Thessaloniki, Greece

**Keywords:** Palmaris longus, Anatomical variations, Presence, Absence, Agenesis, Anatomical anomalies

## Abstract

The prevalence of the palmaris longus (PL) muscle varies more than any other muscle in the human body. Its absence across the world ranges between 1.5% and 63.9%. It presents with many different anomalies, discovered either clinically, intraoperatively or after anatomical examination of cadavers. This paper includes recent studies and reports about the presence and variations of the PL muscle, thereby illustrating the differences between ethnic groups, as well as emphasizing the different ways of finding it, during daily clinical and surgical practice.

## Introduction

The purpose of this review was to investigate published articles concerning the distribution of the palmaris longus (PL) muscle in different populations with a view to highlighting some of the rare anomalies associated with it.

## Literature Search Methods

The research was undertaken electronically to discover all the possible variations of the PL muscle, as earlier reported in researches conducted with different populations. This review contains information from Medline and Google Scholar from 1944 to 2014. The research was made using keywords such as palmaris longus, anatomical variations, presence, absence, agenesis, and anatomical anomalies.

All related articles were carefully assessed and their conclusions and discussions were taken into consideration in this review. Information about unavailable articles was obtained from the published abstract. The published articles that were studied are presented in the discussion section and were all included to the references.

## Literature Results

The search produced many articles on the prevalence of PL muscle in the world. After careful reading, we chose, for our research, 34 articles that contained studies on populations, 20 articles that reported or concluded interesting cases of PL muscle anomalies and two review articles. All the population studies in our research excluded people with previous injury and deformities of the forearm and were made using at least three clinical tests.

## Anatomy

The PL muscle belongs to the superficial flexor muscles of the forearm. The muscles of the forearm are: 1) the anterior or flexors; 2) the posterior or abductors; and 3) the muscles of the radial area. All these muscles are muscular at the upper end and they are toggled into long thin tendons that extend at the apex of the fingers [1]. The PL muscle belongs to the anterior muscles of the forearm. The anterior muscles form three layers, two superficial and one deep. This muscle belongs to the first superficial layer along with the flexor carpi ulnaris, the flexor carpi radialis and the pronator teres muscle [1-3].

The PL muscle is a thin spindle shaped long slender and fusiform muscle that is found between the flexor muscles, carpi ulnaris and carpi radialis [2-6]. It arises from the medial epicondyle and epicondylar ridge of the humerus [3-6]. It runs downwards and terminates in a long, slender tendon which passes anterior to the transverse carpal ligament, crosses the retinaculum, becomes flat and enters the palmar aponeurosis of the hand, anterior to the flexor retinaculum [6-8]. Its innervation comes from branches of the median nerve [1, 3].

## Physiology

The PL muscle flexes the wrist weakly as an accessory flexor muscle. Its main function is to serve as an anchor of the fascia, as it tenses the skin and the palmar fascia of the hand, shearing the forces to the palmar aponeurosis in a distal direction [2, 9-11]. Another use of the muscle is to abduct the thumb [11]. This role is due to its slender move over the long adaptor of the thumb [10-12]. Generally, its role is negligible and this is the reason why it is lost in many operations without cost to the function of the forearm or the wrist [13].

## Clinical Examination

Physical examination of the PL muscle is made using different techniques. Using these sign-techniques, we are able to identify the presence, absence and variations of the PL muscle in the examined person [14-17]. So far, 11 different clinical tests have been referred to in the literature. The most common tests in use are: 1) the standard test or Schaeffer’s test: during this test, the person under examination opposes the thumb to the little finger and then flexes the wrist; 2) Mishra’s I test: the metacarpophalangeal joints of the person’s fingers are passively hyperextended, and then the person is asked to flex the wrist; 3) Mishra’s II test: the person is asked to abduct the thumb against resistance while having the wrist in palmar flexion; 4) Thompson’s test: the person under examination makes a fist and then flexes the wrist while opposing and flexing the thumb over the fingers; 5) Pushpakumar’s test or “two finger sign” method: in this test, the person totally extends the index and middle fingers, while the wrist and other fingers are flexed and the thumb is opposed and flexed over the flexed fingers [14-17].

Compared to those mentioned above, there are six other tests that are rarely used to study the agenesis or existence of the PL muscle [18-20]. These tests are: 1) the Lotus sign test: the person forms a cone shape with all his fingers and thumb, along with wrist flexion and thumb abduction [19, 20]; 2) the four finger sign: the person extends the four fingers with a combination of opposition and flexion of the thumb at the first metacarpophalangeal joint [19, 20]; 3) the open hand technique: the person stretches out all the fingers and extends the wrist slightly [19, 20]; 4) the Cangata test: the person under examination opposes thumb abduction and resists wrist flexion [20]; 5) the Bhattacharya test: the person flexes the wrist against the resistance of the examiner [19, 20]; 6) the Hiz-Ediz test, which is the most recently developed test: the examiner applies resistance to the flexed fingers and the wrist while all the fingers are at the opposite position with the wrist slightly flexed [2, 21].

## Discussion

The PL muscle is one of the most variable muscles in the human body both in percentage and form. The most common anatomical anomaly in the literature is its absence or the so-called agenesis [12, 15, 21]. The traditional knowledge is that PL muscle is absent in 15% of the global population [12]. This information was first reported by Reimann et al (1944) and it is still being constantly questioned by many researchers across the world [12]. Many studies investigated the prevalence of the PL muscle and reported its absence among different populations. All the results from the most recent studies are located in Table 1 according the year of publication.

**Table 1:** Summary of the Percentages of the PL Muscle Agenesis in Each Study [2, 7, 10, 11, 14-26, 28-36, 38-41]

Research	Country - Nation	Number of participants	Agenesis
Offei et al, 2014 [22]	Ghana	210	3.8%
Venter et al, 2014 [32]	South Africa	706	24.4%
Karimi-Jashni et al, 2014 [33]	Iran	732	30.7%
Lahiji et al, 2013 [34]	Iran	1,000	22.8%
Raouf et al, 2013 [18]	Egypt	386	50.8%
Soltani et al, 2012 [35]	USA (Multiethnic)	516	14.9% Caucasians; 2.9% Asians; 4.5% Afro-Americans
Osonuga et al, 2012 [2]	Ghana	226	3.1%
Kyung et al, 2012 [23]	South Korea	269	4.1%
Saxena, 2012 [14]	India	426	27%
Sharma et al, 2012 [7]	India	400	16.25%
Morais et al, 2012 [28]	Brazil	740	26.5%
Kigera et al, 2011 [19]	East Africa	800	4.4%
Ertem et al, 2011 [40]	Turkey	200	34.5%
Alves et al, 2011 [29]	Turkey	200	20%
Hiz, 2011 [21]	Turkey	1,000	15%
Sankar et al, 2011 [30]	India	942	28%
Sater et al, 2010 [26]	Bahrain	1,043	36.8%
Ndou et al, 2010 [36]	South Africa	201	11.5%
Enye et al, 2010 [15]	Lagos	500	12.6%
Eric et al, 2010 [38]	Serbia	800	37.5%
Mbaka and Adedayo, 2009 [10]	Nigeria	600	6.7%
Kose et al, 2009 [11]	Turkey	1,350	26.6%
Gangata, 2009 [20]	Zimbabwe	890	1.5%
Kapoor et al, 2008 [39]	India	500	17.2%
Oluyemi et al, 2008 [16]	Nigeria	600	31.3%
Roohi et al, 2007 [41]	Malaysia	450	11.3%
Sebastin et al, 2006 [17]	China	329	4.6%
Thompson et al, 2001 [31]	Caucasians	300	25%
Ceyhan and Mavt, 1997 [25]	Turkey	7,000	63.9%
Troha et al, 1990 [24]	Caucasians	200	5.5%

There have been 32 studies that have objectively assessed PL muscle presence. The results showed that its absence prevalence ranges between 1.5% and 63.9%. The lowest absence was observed in Zimbabwe (1.5%) with unilateral and bilateral agenesis being 0.9% and 0.6%, respectively [20]. It was also observed that PL muscle agenesis is very low in black populations because of the fact that the lowest percentages were found in Zimbabwe (1.5%) [20], Ghana (3.1-3.8%) [2, 22], East Africa (4.4%) [19] and Nigeria (6.7%) [10]. Low percentages of absence were also found in China (4.6%) [17], South Korea (4.1%) [23] and Caucasians (5.5%) [24]. The highest prevalence of PL muscle absence was observed in Turkey in 1997 by Ceyhan and Mavt that noticed the remarkable percentage of 63.9% of absence [25]. Very high percentages of absence were also found in Egypt (50.8%) [18] and Bahrain (36.8%) [26]. All the other studies that present percentages of absence of the PL muscle in Caucasian, Middle East and Latin American populations were found to have agenesis in percentages between those mentioned before.

The current knowledge is that PL muscle absence is more common in women and on the left side. There have been 10 studies that found significant difference between genders in the PL muscle agenesis. Eight studies concluded that PL absence is more common in females [2, 11, 18, 26-30] and only two studies concluded that PL absence is more common in males [7, 31]. On the other hand, there have been 11 studies that did not find any statistically significant difference between sexes [10, 15, 17, 21-23, 32-36]. According the side of the body where PL muscle absence is more frequent, there have been 23 studies that examined this connection. PL muscle agenesis was more frequent on the left hand in eight studies [2, 25, 29, 30, 34, 37-39] and on the right hand in two studies [27, 31]. In contrast with these results, 13 studies concluded that statistically there is no difference between the two sides of the body [7, 15-18, 21-23, 28, 32, 33, 35, 36].

The PL muscle absence may appear on one side or on both sides of the body. There have been 21 studies that examined the laterality in the PL muscle absence. Bilateral agenesis of the PL muscle was more common in nine studies [11, 16, 18, 25-27, 31, 37, 40] and unilateral agenesis was more common in four studies [7, 19, 38, 41]. On the contrary, eight studies noted that unilateral and bilateral absence of the PL muscle had no statistical difference and concluded that PL muscle agenesis is irrelevant to laterality [17, 20-23, 32, 33, 35].

The PL muscle agenesis is not connected with hand dominance and this incidence was found statistically significant in five studies [17, 21, 23, 27, 33]. There was only one study from Iran where Abdolahzadeh et al in 2013 stressed the fact that there is a strong correlation between PL agenesis and left hand dominance. These authors also stated that people with absence of the PL muscle are 3.7 times more likely to have left hand dominance while left handed people are 3.7 times more likely to have agenesis of the PL muscle [34]. This result was not repeated by any other study.

The PL muscle absence in not correlated with other anomalies of the forearm and there have been four studies that came up to this result [7, 17, 18, 22]. On the contrary, Yesilada et al (2012) reported the case of a young male that was operated and was found to lack the PL muscle in combination with an anomalous flexor digitorum superficialis (FDS) muscle with a large unique belly [42]. Other important conclusions from the literature were the fact that PL muscle absence is not connected with a decrease in the grip or pinch strength of the hand [43] and the fact that PL tendon is rapidly disappearing in humanity [38].

Apart from PL muscle agenesis, which is the most common anomaly of the muscle, there are other variations that concern the form of the muscle, its attachments, origin, course, position, belly or tendon [5, 6, 28, 44]. All these rare anomalies have an overall incidence of 9% and are seen more often on the right hand [5, 6, 28, 44].

There have been 20 interesting articles in the literature from 1975 to 2014 that reported an anomaly of the PL muscle. Ten of these articles referred to intraoperative observations, nine articles referred to anatomical dissections and only one referred to medical records of magnetic resonance imaging (MRI). The results from the anatomical dissections showed that PL muscle may appear unilaterally reversed [3, 45], bilaterally reversed [46, 47], reversed having three bellies [48], unilaterally duplicated [49], bilaterally duplicated [50] and fleshy without the usual distally long tendon [5]. Especially interesting is a study made by Pai et al (2008) who performed anatomical dissections on 30 cadavers and observed that PL muscle was absent in four cadavers while it was reversed in three others [6].

The PL muscle variations can cause symptoms or painful syndromes (carpal tunnel syndrome, Gyon’s syndrome) to the upper extremities that can be relieved only after surgical removal of the muscle. Such kinds of cases are reported rarely. In our research, we found 10 articles that referred to intraoperative discovered anomalies of the PL muscle. There have been two authors that reported Gyon’s syndrome to patients due to a hypertrophied accessory PL muscle [51] and a reversed PL muscle that was directed to the Guyon’s channel [52] respectively. There have been three studies that described anomalous PL muscles in patients that were operated due to carpal tunnel syndrome. In these cases, the PL muscle that was described was either unilaterally reversed (three cases) [53] or hypertrophied and bilaterally reversed [54] or tendinous at its proximal end (1/3), muscular at its distal end (2/3) and terminated in a very short tendon [4]. There have been five cases where patients were operated due to symptoms (pain, paresthesia, numbness, and weakening) on the forearm or the hand. In these cases, the authors reported variations of the PL muscle like absence in combination with an anomalous FDS [42], an epifascial accessory PL muscle [44], a tendinous proximally, fleshy in the middle and tendinous at the upper end PL muscle [8], a three headed reversed PL muscle [55] and a PL muscle that was running anteriorly to the flexor retinaculum before entering the wrist under the surface of the aponeurosis [56]. Finally, the unique article that constituted a retrospective study of medical records of MRI revealed four cases of reversed PL muscles [57].

## Conclusions

The PL muscle has great clinical importance and is classified phylogenetically as a retrogressive muscle [9, 28, 37]. The reasons for its importance are: 1) its tendon is a great landmark to identify the median nerve during operations [5, 44, 49]; 2) the PL tendon is used as an alternative transplant for various reconstructive plastic and hand procedures and as a tendon graft in various positions in otolaryngology and ophthalmology (e.g. lip augmentation, ptosis correction, management of facial paralysis, restoration of lip and chin defects, urinary incontinence, opponensplasty for severe carpal tunnel syndrome and excisional arthroplasty for management of Keinbock’s disease) [7, 12, 14, 31, 33]; 3) PL muscle variations cause a variety of clinical syndromes such as carpal tunnel syndrome, Guyon’s syndrome or compartment syndrome of the forearm or the wrist [3, 48, 52]; and 4) research of the PL muscle helps the understanding of the hereditary of genes responsible for the muscles and their functions [18]. The conclusion from our research is that PL muscle is indeed one of the most variable muscles in the human body and its variations must always be in medical practitioners and surgeons’ mind in order to deal well with symptoms of the forearm in daily practice.
