# Correction: Ioannis Dimitriou et al, Palmaris Longus Muscle’s Prevalence in Different Nations and Interesting Anatomical Variations: Review of the Literature

**DOI:** 10.14740/jocmr2243wc1

**Published:** 2015-12-03

**Authors:** Ioannis Dimitriou, Anastasios Katsourakis, Konstantinos Natsis, Lazaros Kostretzis, Georgios Noussios

**Affiliations:** a Laboratory of Anatomy in the Department of Physical Education and Sports Medicine (Serres), Aristotele University of Thessaloniki, Thessaloniki, Greece; b Department of Surgery, “Agios Dimitrios” General Hospital of Thessaloniki, Thessaloniki, Greece; c Department of Anatomy, Medical School, Aristotele University of Thessaloniki, Thessaloniki, Greece

Corrections to article “Palmaris Longus Muscle’s Prevalence in Different Nations and Interesting Anatomical Variations: Review of the Literature”, by Ioannis Dimitriou et al, published in Vol. 7, No. 11, 2015, p825-830, doi: http://dx.doi.org/10.14740/jocmr2243w. The authors would like to present their names in the format of first name followed by last name, the new author’s list should read as follows.

Ioannis Dimitriou, Anastasios Katsourakis, Konstantinos Natsis, Lazaros Kostretzis, Georgios Noussios

